# A map of evidence using transcranial direct current stimulation (tDCS) to improve cognition in adults with traumatic brain injury (TBI)

**DOI:** 10.3389/fnrgo.2023.1170473

**Published:** 2023-05-12

**Authors:** Julie Lynn Schwertfeger, Charlotte Beyer, Paul Hung, Nathaniel Ung, Caroline Madigan, Alvi Renzyl Cortes, Bharathi Swaminathan, Sangeetha Madhavan

**Affiliations:** ^1^Captain James A. Lovell Federal Health Care Center, United States Department of Veteran Affairs, North Chicago, IL, United States; ^2^Clinical Medicine, Chicago Medical School, Rosalind Franklin University of Medicine and Science, North Chicago, IL, United States; ^3^Department of Foundational Sciences and Humanities, Chicago Medical School, Rosalind Franklin University of Medicine and Science, North Chicago, IL, United States; ^4^Psychiatry Residency Program, Clinical Medicine, Chicago Medical School, Rosalind Franklin University of Medicine and Science, North Chicago, IL, United States; ^5^Chicago Medical School, Rosalind Franklin University of Medicine and Science, North Chicago, IL, United States; ^6^Physical Medicine and Rehabilitation, Captain James A. Lovell Federal health Care Center, North Chicago, IL, United States; ^7^PM&R Residency Program, Clinical Medicine, Chicago Medical School, Rosalind Franklin University of Medicine and Science, North Chicago, IL, United States; ^8^Rehabilitation Sciences Program, and Physical Therapy Program, University of Illinois Chicago, Chicago, IL, United States

**Keywords:** rehabilitation, neuroimaging (anatomic), dosage accuracy, neuromodulation, transcranial electric stimulation (TES), non-invasive brain stimulation (NIBS), realistic volumetric approach-based simulator for transcranial electric stimulation (ROAST), NINDS common data elements

## Abstract

**Introduction:**

Cognition impairments often occur after a traumatic brain injury and occur at higher rates in military members. Cognitive symptoms impair daily function, including balance and life quality, years after the TBI. Current treatments to regain cognitive function after TBI, including medications and cognitive rehabilitation, have shown limited effectiveness. Transcranial direct current stimulation (tDCS) is a low-cost, non-invasive brain stimulation intervention that improves cognitive function in healthy adults and people with neuropsychologic diagnoses beyond current interventions. Despite the available evidence of the effectiveness of tDCS in improving cognition generally, only two small TBI trials have been conducted based on the most recent systematic review of tDCS effectiveness for cognition following neurological impairment. We found no tDCS studies that addressed TBI-related balance impairments.

**Methods:**

A scoping review using a peer-reviewed search of eight databases was completed in July 2022. Two assessors completed a multi-step review and completed data extraction on included studies using a priori items recommended in tDCS and TBI research guidelines.

**Results:**

A total of 399 results were reviewed for inclusion and 12 met the criteria and had data extracted from them by two assessors using Google Forms. Consensus on combined data results included a third assessor when needed. No studies using tDCS for cognition-related balance were found.

**Discussion:**

Guidelines and technology measures increase the identification of brain differences that alter tDCS effects on cognition. People with mild-severe and acute-chronic TBI tolerated and benefited from tDCS. TBI-related cognition is understudied, and systematic research that incorporates recommended data elements is needed to advance tDCS interventions to improve cognition after TBI weeks to years after injury.

## Introduction

Over 1.5 million TBIs occur in the US annually, of which 75% are mild, with 40% of mild TBI (mTBI) cases having chronic symptoms (Ingebrigtsen et al., 1998; Gerberding and Binder, 2003). Diffuse white and gray matter damage occurs in TBI and this impairs the information relay needed for cognitive function (Andriessen et al., 2010; Dixon, 2017). Persistent cognitive impairments have been reported months to more than 10 years after TBI (Draper and Ponsford, 2008). Attention deficits are reported in 48% of adults with subacute TBI, 38% of those with chronic mild TBI, and up to 50% of adults with chronic moderate-to-severe TBI (Parker et al., 2005; Draper and Ponsford, 2008; McFadyen et al., 2009; Parrington et al., 2020; Tsai et al., 2021).

Current treatments for cognition after TBI, including drug and cognitive rehabilitation, have limited efficacy. Drug treatments for cognition after TBI provide inconsistent cognitive improvements and significant burdens and side effects (Xiong et al., 2009; Dougall et al., 2015). Similarly, cognitive rehabilitation interventions have limited benefits and often do not translate to daily life (Hallock et al., 2016; Cicerone et al., 2019).

Transcranial direct-current stimulation (tDCS) is a form of non-invasive brain stimulation (NIBS) that is safe and effective for improving cognitive functions in adults (Dong et al., 2021; Pol et al., 2021; Cammisuli et al., 2022). Moreover, combined with existing treatments, tDCS improves functional outcomes. Though not approved for clinical use, tDCS is considered a minimal risk by the Food and Drug Administration for use in people with neurological impairments as a result of psychiatric symptoms such as depression (Sánchez-Kuhn et al., 2017; Boissonnault et al., 2021).

Limited but promising evidence supports tDCS as safe and effective for cognition across all levels of TBI acuity and severity (Begemann et al., 2020). The high prevalence of people struggling with persistent TBI-related cognitive deficits, the paucity of studies investigating tDCS for cognition after TBI, and the recently updated guidelines that identify methods and data elements to advance science and applications for TBI tDCS studies provide the need for data elements to map the current evidence for using tDCS for cognition after TBI. A map of studies using tDCS for cognition after TBI, based on guideline-recommended elements will facilitate discovery and translation to improve rehabilitation outcomes (Grove et al., 2013; Fregni et al., 2015; Lefaucheur et al., 2017; Thair et al., 2017; Vyvere et al., 2020; LaPlaca et al., 2021).

In this study, we performed a systematic scoping review to map current studies of tDCS to improve cognition for adults with TBI. The map used guideline-recommended items for the safety and reproducibility of tDCS and TBI studies to advance clinical translation research and rehabilitation outcomes after TBI.

## Methods

The objective was to map existing tDCS studies for cognition or balance in adults with TBI. The scoping review format was selected based on the relative absence of TBI randomized controlled trials (RCT) in systematic reviews of tDCS for cognition in neurologic populations and the absence of tDCS RCTS using tDCS for balance after TBI.

The search strategy aimed to locate both published and unpublished studies. An initial limited search of MEDLINE (PubMed) was undertaken to identify articles on the topic using controlled vocabularies such as MeSH (Medical Subject Headings) as well as relevant keywords. The search strategies used in this review were designed and executed by a health sciences librarian (CB) and verified by a content expert (JS). Keywords and controlled vocabulary terms located in article titles, abstracts, and other fields in the records within the search results were analyzed to determine the word set used in the final search strategy. In addition to search terms, this search incorporated search fields. Since tDCS is a specialized topic, the decision was made to use the text word field instead of restricting it to the title and abstract field. By utilizing the text word field, we cast the widest net for resources with tDCS in the article title, abstract, journal title, article keywords, and throughout the full article text. The search filter was used to limit retrieval by the English language due to the linguistic limitations of the author team. The PubMed search strategy was translated to be able to retrieve information from a variety of sources.

Searches were adapted to each database's particular syntax, search fields, index terms, and keywords to maintain sensitivity and specificity across databases. The information resources were originally searched to locate studies included: CINAHL Complete (EBSCO), Cochrane Library, Embase (Elsevier), PEDro, PsycINFO (EBSCO), JBI Evidence Synthesis (Ovid), JBI Evidence Implementation (Ovid), and Web of Science. Searches for unpublished studies and gray literature were made in Google Scholar. Key websites of Neuromodec (https://neuromodec.org/) and the International Neuromodulation Society (https://www.neuromodulation.com/) were also searched. The team also reviewed reference lists of key articles to identify additional resources. The initial search used the concepts TBI, t-DCS, and balance. However, limiting the search to those concepts resulted in minimal results. The authors discussed the results and used input from content experts to expand the search to include the specific cognitive domain terms of learning, memory, balance, executive function, problem-solving, and attention. The search strategy for MEDLINE (PubMed) is included in Appendix 1.

Search results were uploaded to RefWorks, a citation management system, and reviewed for inclusion criteria by two independent assessors (JS, PH) (Tricco et al., 2018). Consensus guidelines for tDCS and for TBI research were used to determine a priori data extraction items (JS), and a second assessor (AC) reviewed the extraction item list for coherence with the guidelines. A form was created to extract article data (JS), which was later piloted and revised (JS, PH, NU, CM). Two independent assessors (NU, CM) extracted and combined the results, and assessors (NU, CM, JS) achieved consensus.

## Results

The search returned 399 citations of which 12 met the criteria for inclusion. The Preferred Reporting Items for Systematic Reviews and Meta-Analysis scoping review flowchart showing the review stages is available as Appendix 2 (Moher et al., 2009).

Included studies, listed in Table 1, represent research from Australia, Canada, Italy, South Korea, Poland, and the USA published between 2012 and 2021, all using anodal tDCS (A-tDCS) and targeting default mode attention and salience networks or networks connecting to these (Kang et al., 2012; Leśniak et al., 2014; Ulam et al., 2015; Sacco et al., 2016; O'Neil-Pirozzi et al., 2017; Li et al., 2019; Motes et al., 2020; Quinn et al., 2020; Boissonnault et al., 2021; Chiang et al., 2021; Eilam-Stock et al., 2021). The studies include people with mild to severe TBI, acute through chronic recovery phases, aged from 21 to 69 years and belonging to both male and female genders. Sample sizes ranged from one (Leśniak et al., 2014; Eilam-Stock et al., 2021; Rushby et al., 2021) (Kang, Eilam-Stock, Chiang) to 59 participants (Li et al., 2019). Ten studies used traditional tDCS and two used high-definition tDCS (HD-tDCS) (Motes et al., 2020; Chiang et al., 2021). Eight studies were RCT with single- or double-blinding methods, two were single-case designs, of which one was a prospective single-blind pilot and the other was an uncontrolled pilot study. Guideline items for tDCS and TBI reporting in studies were good to strong for all tDCS items except for medications, handedness, confirmed dose delivered, and adverse events. All but one study reported the task that participants performed during tDCS and only one reported the day and time of tDCS sessions.

**Table 1:** Key characteristics of tDCS studies for cognition after TBI.

**Lead author, *N*_TBI_, age, % female, TBI acuity, severity**	**Study type**	**Yrs edu-cation**	**MRI**	**Brain target**	**Elec-trodes**	**A**	**C**	**Map**	**mA**	**Dose**	**Device name**	**Behavi-or target**	**Physiologic target**	**Results**
Kang, *N* = 9, 20–78 y/o, (12.5% F), Chronic, ^*^moderate-to-severe TBI	RCT, double blind	NR	N	LDLPFC	5 × 5 cm	F3	Fp2	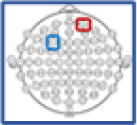	2.0	20 min, 1 session	Phoresor^®^ II PM850, IOMED	A, EF, RT	Excite TBI damaged frontal lobes to ↑ function	↓ reaction time 1 session A-tDCS not after sham
Leśniak, *N* = 23, 18–45, (35% F), subacute-to-chronic, severe TBI	RCT, double blind	14.5	Y	LDLPFC	5 × 7 cm	F3	Fp2	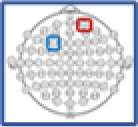	1.0	10 min, 15 sessions, 1 × /day	Eldith, 1 Ch DC Plus Neuroconn	A, RT	Excite to promote LTP for learning during Cog Rehab	↑ selective and sustained attention in tDCS vs. sham group
Ulam, *N* = 26, 18–65, (15.3% F), acute–subacute moderate-to-severe TBI	RCT, double blind	9.8–14.3	Y	LDLPFC	5 × 5.6 cm	F3	Fp2	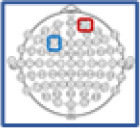	1.0	20 min, 10 sessions, 1 × /day	Magstim, Eldith DC, Neuroconn	A, IS, WM	EEG Δ biomarker to guide TBI tDCS to brain targets	↓ delta, ↑ alpha on EEG A-tDCS group correlated with ↑cognitive function
Sacco, *N* = 32, 18–66, (19% F), chronic, severe TBI	RCT, single blind	11.5 SD 3.48	Y	R or L DLPFC	5 × 7 cm 5 × 7 cm	F3, F4	Arm	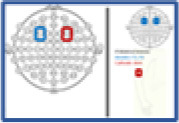	2.0	20 min, 10 sessions, 2 × /day	HDCstim, Newronika srl	A, EF, LM, RT	fMRI Δ - biomarker of normalized limbic function	↑ divided attention and reaction time, ↓fMRI Inf frontal gyri in tDCS group
O'Neil-Pirozzi, *N* = 8, 33–53, (50% F), chronic, severe TBI	RCT, blind-ing NR	13 SD 2	Y	LDLPFC	A-5 × 7 C-4 × 5 cm	F3	Fp2	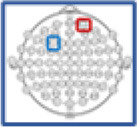	2.0	20 min, 1 session	Geodesic Sensor, El. Geodesics	WM	EEG Δ biomarker of↑ working memory after tDCS	↑ word recall A-tDCS, not C-tDCS or sham. ↑EEG Oddball task C-tDCS only
Li, *N* = 59, 21–56, (14.3% F), chronic, moderate-to-severe TBI	RCT, blind-ing NR	NR	Y	RiFG/aIC	A-4.5 cm, C-7.5 cm	F8	Neck	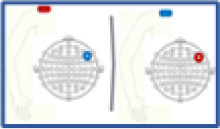	2.0	5 min, 1 session,	Neuroconn	A, I/S, WM	fMRI, EEG Δ, Default mode, salience network	Poor Stop Signal Task with ↑ white matter damage at post CG / mPFC
Motes, *N* = 14, 35–50, (7%F), chronic, ^*^mild-to-moderate TBI	Prospective, no blind	14.5	N	Pre SMA/dACC	1 cm Ag/AgCl	FZ	Fp1, Fp2, F7, F8	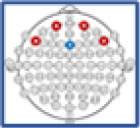	1.0	20 min, 10 sessions, 1 × /day	Starstim tCS^®^, Neu -roelectrics	A, EF, L/M,	Salience network. Thalamic, DMN connection	↑ word finding, fluency, abstract thought, decision making 8 weeks post A-tDCS
Quinn, *N* = 24, 18–59, (37.5% F), chronic, mild-to-moderate TBI	RCT, double blind	14.7	N	LDLPFC	5 × 5 cm	F3	R arm	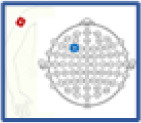	2.0	30 min, 10 sessions, 1 × /day	NeuroConn tDCS, neuroCare	A, EF, mood	Blood flow Δ using Pseudo-arterial spin labeling	↑ or stable cerebral blood flow inf frontal gyrus A-tDCS group, not sham
Boissonnault, *N* = 6, 49–74, (17% F), acute-to-subacute, mild complex–severe TBI	Pilot, no control	NR	Y	LDLPFC	5 × 5 cm	F3	Fp2	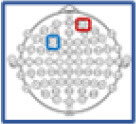	2.0	20 min, 9 sessions, 3 × /week	Soterix tDCS (1,300 A)	A, utility	Feasibility of tDCS for attention subacute TBI	A-tDCS tolerated subacute TBI, no major AEs, poor (< 50%) completion
Chiang, *N* = 1, 39 y/o F chronic, severe TBI	Case study	NR	Y	Pre SMA/ dACC	1 cm Ag/AgCl	FZ	Fp1, Fp2, F7, F8	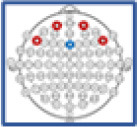	1.0	20 min, 10 sessions, 5 × /week	Neuroelet-rics, Starstim^®^	A, I/S, L/M	cortico-caudate-thalamic (word finding) circuit	HD-tDCS ↑ verbal fluency, naming, working memory, exec function × 14 weeks
Eilam-stock, *N* = 1, 29 y/o M, chronic, moderate TBI (remotely supervised tDCS)	Case study	NR	N	LDLPFC	5 × 5 cm	F3	F4	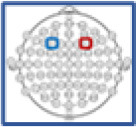	2.0	20 min, 20 sessions, 1 × /day	Soterix Medical, mini-CT	A, L/M, RT, mood	Remote supervised home tDCS (RS-tDCS) w/ cog rehab	rtDCS ↑ working memory, verbal fluency, processing speed > 1 SD, ↑mood
Rushby, *N* = 30, 21–69, (23%F), chronic, severe TBI	RCT, single blind	12.7	Y	Left Parietal Cortex	5 × 7 cm	P3/P4	P4/P3	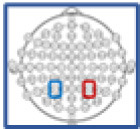	2.0	20 min, 1 session	Eldith, NeuroConn	RT, WM	Parietal access to DMN/ salience network post TBI	1 × A-tDCS ↑ reaction time correlated with ↓ task arousal (skin Ω), no Δ WM

“^*^” TBI severity at the time of injury is based on text descriptions in the study and without a specific TBI severity scale. Anatomical targets: LDLPFC, left dorsolateral prefrontal cortex; RiFG/aIC, right inferior frontal gyrus/ anterior insula; Pre SMA/dACC, presupplementary motor area/dorsal anterior cingulate cortex. A, attention; EF, executive function; I/S, inhibition/suppression; L/M, learning/memory; RT, reaction time; WM, working memory; DMN, default mode network; postCG/mPFC, posterior cingulate gyrus/medial prefrontal cortex; rtDCS, remotely supervised tdcs.

All studies reported many recommended TBI items, shown in Table 2, including time since TBI and initial severity. Most studies reported TBI lesion size and location and years of education. Less than half of the studies reported race and ethnicity. Very few studies reported participant marital status, household income, people in household, history of seizure or post-traumatic amnesia (PTA), physical function level, or work status.

**Table 2:** Review of tDCS guidelines and TBI common data element items in tDCS studies for attention/cognition after TBI.

**Consensus guideline checklist for published papers using tDCS for attention after TBI**																			**TBI NINDS common data elements**												
**References**	**Age**	**Gender**	**Handedness**	**Medication**	**Nicotine/caffeine/alcohol**	**Include/exclude criteria**	Confirmed dose delivered*	**Type of stim**	**Primary OM**	**Stim Intensity and Duration**	Adverse Events (AEs)*	**Electrode type**	**# of electrodes**	**Target electrode**	**Return electrode**	**Task during stim**	Session day/time*	**Study duration**	**TBI severity—acute**	**TBI cog severity now**	**Function (GOSE/FIM/EBIQ/mRS)**	**Time since TBI**	**PTCS or PTA after TBI** ^*^	**Seizure**	**Lesion size and location(s) load/location**	**Household income** ^*^	# People in household*	**Marital status** ^*^	**School years/degree(s)**	**Ethnicity and race**	**Work status** ^*^
Kang et al. (2012) (KR)	X	X	–	–	–	X	–	X	X	X	–	X	X	X	X	–	–	X	–	X	–	X	–	X	X	–	–	–	–	–	–
Leśniak et al. (2014) (PL)	X	X	–	–	–	X	–	X	X	X	?	X	X	X	X	–	–	X	X	–	X	X	–	X	X	–	–	–	X	–	X
Ulam et al. (2015) (US)	X	X	–	X	X	X	–	X	X	X	–	X	X	X	X	X	–	X	X	X	X	X	–	–	X	–	–	–	X	X	–
Sacco et al. (2016) (IT)	X	X	–	–	–	X	–	X	X	X	?	X	X	X	X	X	–	X	X	X	–	X	–	–	X	–	–	–	X	–	–
O'Neil-Pirozzi et al. (2017) (US)	X	X	X	X	–	X	–	X	X	X	X	X	X	X	X	–	X	X	X	–	–	X	–	–	X	–	–	–	X	–	–
Li et al. (2019) (UK)	X	X	–	–	–	X	X	X	X	X	–	X	X	X	X	X	–	X	X	–	–	X	–	–	X	–	–	–	–	–	–
Motes et al. (2020) (US)	X	X	X	–	X	–	X	X	X	X	?	X	X	X	X	X	–	X	X	–	–	X	–	–	–	–	–	–	X	X	–
Quinn et al. (2020) (US)	X	X	X	X	X	X	–	X	X	X	?	X	X	X	X	X	–	X	X	–	X	X	?	X	–	–	–	–	X	–	–
Boissonnault et al. (2021) (CA)	X	X	–	–	–	X	–	X	X	X	X	X	X	X	X	X	–	X	X	X	–	X	–	X	X	–	–	–	X	X	–
Chiang et al. (2021) (US)	X	X	X	X	–	–	X	X	X	X	–	X	X	X	X	X	–	X	X	X	X	X	X	X	X	–	–	–	X	X	X
Eilam-Stock et al. (2021) (US)	X	X	X	–	–	–	–	X	X	X	–	X	X	X	X	X	–	X	X	–	–	X	–	–	–	–	–	–	–	X	–
Rushby et al. (2021) (AU)	X	X	X	X	X	X	–	X	X	X	–	X	X	X	X	X	–	X	X	–	–	X	X	X	X	X	–	–	X	–	–

“^*^”denoted that 25% or fewer of included studies reported this in manuscript and or supplements; “?” denotes that authors stated this was addressed, without details to confirm whether it met guidelines; “X” denotes the inclusion of people with a history of this (seizure, PTA).

### Anatomic targets and electrode placements

Studies reported brain targets for stimulation and electrode placement using standardized terminology: the anode, the negative electrode, is stated as A and the cathode, the positive electrode, is stated as C. Electrode locations on the skull are stated using standardized 10–20 electroencephalogram map letter-number coordinates. The amount of current delivered was stated in milliamps (mA).

Eight studies targeted the dorsolateral prefrontal cortex (DLPFC), seven targeted the left (LDLPFC), and one targeted the lesioned DLPFC. Five studies targeting the LDLPFC (Kang et al., 2012; Leśniak et al., 2014; Ulam et al., 2015; O'Neil-Pirozzi et al., 2017; Boissonnault et al., 2021) used electrode placements A-F3, C-Fp2. A sixth study (Eilam-Stock et al., 2021) used A-F3 but differed by placing C-F4 corresponding to the right DLPFC. Similarly, the seventh study (Sacco et al., 2016) targeted the damaged DLPFC so that electrodes were placed at A-F4, C-F3 for people with right hemisphere damage, at A-F3, C-F4 for people with left hemisphere damage, and at A-F3, A-F4, C-on the arm for people with bilateral brain lesions. The eighth study (Quinn et al., 2020) used A-F3 and C-right arm sub-deltoid based on computer modeling to maximize current density at the LDLPFC.

Four studies placed electrodes based on current flow computations (Li et al., 2019; Motes et al., 2020; Quinn et al., 2020; Chiang et al., 2021). The first study (Quinn et al., 2020) used A-F3 and C-right sub-deltoid to restrict current from beyond the LDLPFC in the brain. The second study (Li et al., 2019) used computer models to identify A-F8, C-right neck base targeting the salience network node at the right inferior frontal gyrus/anterior insula. The other two studies (Motes et al., 2020; Chiang et al., 2021) used HD-tDCS, with A-FZ corresponding to the location of the thalamic connection to the default mode network and salience network node and C-F7, Fp1, F8, and Fp2 targeting the supplementary motor area and the dorsal anterior cingulate gyrus cortico-caudate-thalamic circuit.

### Timing and pairing with behavioral interventions

#### Studies of people undergoing TBI rehabilitation including people < 6 months post-TBI

Study populations varied by TBI acuity and whether tDCS was paired with a cognitive intervention. Three studies took place during rehabilitation, of which two (Ulam et al., 2015; Boissonnault et al., 2021) included people with acute to subacute complex-mild-to-severe TBI, and a third (Leśniak et al., 2014) included subacute and chronic severe TBI. These studies also varied regarding behavioral interventions paired with tDCS. All these studies used traditional A-tDCS to A-F3, C-Fp2 to stimulate the LDLPFC.

The Boissonnault study (Boissonnault et al., 2021) was an unblinded feasibility pilot study on six people with acute-subacute complex-mild-to-severe TBI aged 49–74 years undergoing specialized TBI inpatient rehabilitation. The intervention delivered nine 20-min sessions of A-tDCS at 2.0 mA. The study aimed to identify a protocol using 3 × /week tDCS during inpatient rehabilitation. Patients continued “regular function rehabilitation”, the details of which were not reported in the study. The findings were that patients found the treatments beneficial and session completion rate was low. Barriers identified to using tDCS during inpatient rehabilitation included patient scheduling, early discharge, and contraindications for tDCS.

The Lesniak study (Leśniak et al., 2014) was a pilot single-blinded RCT on patients aged 18–45 years undergoing inpatient or outpatient rehabilitation for memory impairment 4–92 months after severe TBI. The study assessed whether a tDCS intervention that improves memory in healthy adults was effective for people with TBI (15 10-min A-tDCS using 1.0 mA paired with computerized cognitive rehabilitation on memory). Of the participants, 12 people receiving A-tDCS had greater improvements in memory and attention tests that were not statistically significant as compared to the sham group, which did not support the intervention parameters used in healthy controls for memory translation due to severe TBI did not support the intervention parameters used in healthy controls.

The Ulam study (Ulam et al., 2015) was a double-blinded RCT that involved 26 people aged 18–65 years with acute-subacute moderate-severe-TBI that used 10 20-min sessions of 1.0 mA A-tDCS (*n* = 13) vs. sham (*n* = 13) paired with EEG. The study's aim was to demonstrate that increased alpha and decreased delta oscillations following A-tDCS would correspond to improved memory and attention. The A-tDCS group showed EEG changes, but the sham group did not. Participants with the greatest amount of baseline slow-wave activity had the largest changes in cognition and EEG post-tDCS.

#### Studies of people with chronic TBI (>6 months post TBI)

Our search identified nine studies using tDCS for cognition in people more than 6 months post-TBI. These studies included participants with a range of injury severity, of which seven included people with moderate-to-severe TBI and two included people with mild-to-moderate TBI. The methods and aims used in these studies ranged from single or multiple tDCS sessions to tDCS sessions paired with a cognitive-behavioral intervention and varied in electrode placement and tDCS parameters.

#### Studies using a single tDCS session without a behavioral intervention for people with chronic TBI

A double-blinded crossover pilot study of nine people with moderate-to-severe-TBI aged 20-78 years was completed by Kang et al. (2012). This study assessed whether a single 20-minutes A-tDCS session of 2.0 mA to A-F3, C-Fp2 improved attention. The study results were increased reaction times immediately after the A-tDCS condition that were not retained 3 h later. This study also found that improved reaction time did not occur following the sham tDCS condition. The O'Neil-Pirozzi et al. (2017) pilot RCT study on four people with chronic-severe-TBI and four healthy controls used one session of 2.0 mA A-F3, CFp2 and resulted in improved verbal recall for both groups and also improved EEG results in the TBI group. The Rushby et al. (2021) study included 30 people aged 21–69 years with chronic severe TBI and compared the effects of 2.0 mA A-TDCS to the left parietal cortex A-P4, C-P3 versus sham on working memory and skin conductance (a measure of task-specific arousal). Rushby et al. (2021) argued that the parietal brain is relatively undamaged after TBI whereas the DLPFC area is usually damaged after TBI and both placements stimulate the same attention network. The study results did not support that a single dose of tDCS using these settings improved working memory in people with chronic severe TBI.

An RCT (Li et al., 2019) of 35 people with moderate-to-severe TBI and 24 healthy controls aged 21–56 years investigated EEG and fMRI as biomarkers of cognition improvement. Specifically, fMRI, EEG, and complex attention task performances were assessed during each of the A-tDCS session of 2.0 mA to A-F8, C-neck base vs. cathodal or sham. Significant improvements in response suppression resulted from A-tDCS in the controls but not those with TBI. Li's study identified that people with tissue damage in connections between the right inferior gyrus and the anterior insula had lower scores on the attention task.

#### Studies providing multiple tDCS sessions without a behavioral intervention

A single-blind prospective study Motes et al. (2020) provided 10, 20-min sessions of 1.0 mA tDCS to eight veterans and sham stimulation to six veterans. All participants had chronic TBI, were aged between 35 and 50 years, and likely had complex mild-to-moderate TBI based on imaging. The A-tDCS group significantly improved in word recall and the improvement remained weeks later.

The Chiang et al. (2021) case study paired A-HD-tDCS targeting pre-SMA/dACC and identified normalized event-related potentials on EEG that correlated with cognitive improvements in a 39-year-old woman 3 years post-severe chronic TBI with prolonged loss of consciousness, post-traumatic amnesia, and seizure that was now resolved. This study provided 1.0 mA for 10 20-min sessions. The patient had problems concentrating, finding words, remembering names, recognizing people, and switching attention, which prevented her from returning to work as an executive. After the intervention, her working memory, word-finding, and executive functions improved, and her EEG event-related potentials were normalized. The improvements were maintained 14 weeks after the end of treatment.

#### Studies using multiple tDCS sessions paired with a behavioral intervention

Three studies on people with chronic TBI (Sacco et al., 2016; Quinn et al., 2020; Eilam-Stock et al., 2021) paired A-tDCS with computerized cognitive rehabilitation.

Sacco et al. (2016) studied fMRI as a biomarker for improved cognition in a single-blind RCT of 32 people (16 active, 16 sham) with severe-chronic-TBI aged 18–66 years and used 10 20-min sessions of 2.0 mA A-tDCS vs. sham to the damaged DLPFC. The A-tDCS group had increased divided attention, reaction time, and decreased inferior frontal gyrus activation, which supported existing evidence that A-tDCS normalized inferior gyrus activation and that normalized frontal gyrus function corresponded with improved attention on behavioral tests.

The Quinn et al. (2020) pilot RCT of 24 (10 active, 14 sham) participants with mild-to-moderate TBI used a 30-min computerized executive function training for 10 days simultaneously with 30-min A-tDCS at 2.0 mA to A-F3 and C-right sub-deltoid or sham. This study also used MRI pseudocontinuous arterial spin-labeling to measure changes in cerebral blood flow (CBF). The A-tDCS group had stable or increased CBF in the rIFG whereas CBF decreased the sham group. However, CBF changes did not correlate with neuropsychological test changes.

The Eilam-Stock et al. (2021) case study assessed remotely supervised tDCS on a 29-year-old man 4 years post-moderate TBI who self-administered 20 sessions of A-tDCS at 2.0 mA to A-F3, C-F4 at home paired with computer cognitive rehabilitation (Brain-HQ^®^) exercises under video supervision. The patient's symptoms included impaired information processing, focus, memory, impulse control, and mood that impaired his success at work. The patient improved more than one standard deviation in tests of attention, verbal fluency, working memory, and information processing speed. His mood also improved.

## Discussion

This review mapped 12 post-TBI tDCS cognition studies. Promising results from limited studies for each TBI acuity and severity level support the need for further studies. Further, many studies were underpowered for acute-to-subacute TBI for each severity level, which is when most expected recovery and rehabilitation services are provided. Differences in methods prevent combining results of the few RCTs for chronic TBI. Despite adequate design methods, current tDCS research for TBI-related cognitive impairments provides only preliminary insights supporting that tDCS may improve physiologic and behavioral cognitive function after TBI. Importantly, these results support that it is safe, inexpensive, and prudent to further research the use of tDCS for cognition across all TBI acuities and severities using biomarkers including electrical-field modeling (Evans et al., 2020; Molero-Chamizo et al., 2021; Mizutani-Tiebel et al., 2022; Nasimova and Huang, 2022).

The aims of these studies ranged from establishing inpatient protocols, tDCS safety for severe TBI and seizure history, biomarkers for treatment and outcome prediction, to replicating non-TBI designs in a TBI population. This broad range of aims limits depth for any single aim. This result underscores the need for further research with common aims, in populations with similar characteristics, using similar methods and measures.

A trend in this map constitutes methods to delimit the current path, tailor dosage, and link biomarkers to behavioral outcomes. Positive results support that brain changes from tDCS underlie behavioral outcomes. As a group, they support further research using computer modeling for treatment and identification of outcome differences from tDCS across participants with TBI and in general. This map also presents an opportunity for tDCS studies on people with TBI to incorporate the NINDS CDEs, which were established to advance research of this under-researched diagnosis using common data items and inviting researchers to add information into the Federal Interagency Traumatic Brain Injury Research (FITBIR) Informatics System, which facilitates data-sharing and collaboration between studies (Moher et al., 2009; Grove et al., 2013; Ivory, 2015; Tricco et al., 2018; Vyvere et al., 2020).

Limited but promising evidence supports that tDCS may improve cognition after TBI at all acuities and severities. The value of advancing this research is depicted in two cases highlighting the financial and life-quality costs of the current gaps in care for post-TBI impairment. Both cases provided tDCS combined with cognitive rehabilitation to working-age adults years after their TBI injury. Both participants struggled with TBI-related cognitive impairments that impaired daily functioning and their ability to work. Following treatment, both participants had significant improvements in cognition, daily function, and mood. One case also identified that the behavioral improvements corresponded with brain EEG changes. Further research is needed to establish tDCS interventions for TBI-related cognitive impairments (Chiang et al., 2021; Eilam-Stock et al., 2021).

## Author contributions

JS contributed to all aspects of this manuscript and led this project. CB contributed to the methods, peer review and revisions to the search strategy, completing the searches, and manuscript review. PH contributed to the concept, introduction, article inclusion and exclusion assessment stages, table creation, and manuscript review. CM and NU contributed to included article extractions and manuscript and review and revision. AC assisted in review of background articles and reviewed the items listed in tables for coherence with referenced guidelines. BS contributed to the concept and manuscript review. SM contributed to the concept, search parameters, and manuscript review. All authors contributed to the article and approved the submitted version.

## Glossary

**A, Anode or anodal**.: This is the negative electrode. Within certain parameters used in transcranial stimulation, anodal stimulation makes the neurons more likely to fire by bringing the neuronal membrane closer to depolarizing.
**C, Cathode or cathodal**.: This is the positive electrode. Within certain parameters, cathodal stimulation decreases the likelihood that a neuron is more likely to fire by hyperpolarizing the neuronal membrane.
**A-tDCS, Anodal transcranial direct current stimulation**.: This refers to studies that place the anode over the brain target to stimulate it. Typically, A-tDCS increases excitability.
**C-tDCS, Cathodal transcranial direct current stimulation**.: This refers to placing the cathodal electrode over the brain target. Typically, cathodal stimulation decreases excitability.
**DLPFC, Dorsolateral Prefrontal Cortex**,: the anatomic location on the right and left brain hemisphere.
**EEG, Electroencephalogram**.: This is a study of waveforms between different points in the brain that are measured by placing surface electrodes on specific points around the skull and face.
**ERP, Event-related potential**.: EEG also measures event-related evoked potential, which are measured changes in EEG that result from stimuli and changes in cognition
**fMRI, Functional magnetic imaging**.: This provides detailed images of brain structures over time while the person performs a task. It is used t identify brain structures and functioning related to activities and are also used to identify brain structures that underly treatments and their benefits.
**HD-tDCS, High-definition transcranial direct current stimulation**.: This is a form of tDCS that improves the ability to concentrate current on the target of interest and reduce current from going to brain and nervous system structures outside of the target. HD-tDCS uses multiple return electrodes are used to reduce the current strength of exiting current and to better constrain current density to a limited area and set of brain structures.
**LDLPFC**,: Left DLPFC (on the left-brain hemisphere).
**mA, Milliamps**.: A measure of current strength used in tDCS treatment and other electrical interventions.
**MRI, Magnetic resonance imaging**.: Imaging that is used in tDCS studies to see details of brain structures and fluids. MRIs are created using high-frequency radio waves within a strong magnet that the person lies in. During MRI, the atoms within cells are aligned using different radio-frequencies to best image different types of structures.
**NIBS, Non-invasive brain stimulation**.: This is an umbrella term that encompasses any brain stimulation technique that does not require piercing skin or other body tissues or structures on the person receiving the stimulation.
**NINDS CDE, National Institutes of Neurological Disorders and Stroke Common Data Elements**.: This is a set of data items, measures, and formatting standards developed by subject matter experts to improve the ability to combine study results and interpret results across studies with the aim of accelerating discovery and scientific advances.
**RDLPFC**,: Left dorsolateral prefrontal cortex. Right DLPFC (on the right-brain hemisphere).
**RiFG / dACC, Right inferior frontal gyrus/dorsal anterior cingulate cortex / dorsal anterior cingulate cortex**.: The area on the right hemisphere of the brain where the inferior gyrus and the dorsal aspect of the cingulate gyrus intersect. This area is determined, based on studies, to be the site where stimulation can reach the salience network connections to test connectivity and / or to provide an intervention to this network.
**RiFG/aIC, Right inferior frontal gyrus/anterior insular cortex**.: The anterior insula overlays how we feel or interpret meaning from a stimulus. Also, t's location places it within multiple cognitive and salience networks, which makes it a target used in some NIBS studies.
**TBI, Traumatic brain injury**.: An injury to structures or physiologic processes in the brain that is caused from an external mechanism, which could include blast waves, rapid acceleration/ deceleration, being struck by or striking into an object.
**tDCS, Transcranial direct current stimulation**.: This is a specific form of NIBS that uses surface electrodes and a conductive medium (saline solution or saline gel) to provide direct current into the brain.
