# Supplementary material for: A map of evidence using transcranial direct current stimulation (tDCS) to improve cognition in adults with traumatic brain injury (TBI)
Source: Front Neuroergon. 2023 May 12;4:1170473. doi: 10.3389/fnrgo.2023.1170473 (PMC10790940; doi:10.3389/fnrgo.2023.1170473)
Supplement: Supplementary file 1 [file Data_Sheet_1.pdf]

Appendix 1. MEDLINE (PubMed) Search

PubMed Search		
Search #	Term	# Results
1	(((T-DCS[Text Word]) OR (TDCS[Text Word])) OR ("Transcranial Direct Current Stimulation"[Text Word])) OR ("Transcranial Direct Current Stimulation"[MeSH Terms])	7,571
2	(((((tbi[Text Word]) OR (Traumatic Brain Injur*[Text Word])) OR (Brain injur*[Text Word])) OR (Brain trauma[Text Word])) OR (concussi*[Text Word])) OR (brain injuries, traumatic[MeSH Terms])	119,669
3	(Balanc*[Text Word]) OR (Postural balance[MeSH Terms])	390,322
4	(((Attention[Text Word]) OR (Attention focus[Text Word])) OR (Focus of attention[Text Word])) OR (attention[MeSH Terms])	549,898
5	((Cognition[Text Word]) OR (Fluid cognition[Text Word])) OR (cognition[MeSH Terms])	302,627
6	(executive function*[Text Word]) OR (executive function[MeSH Terms])	39,845
7	(((((learn*[Text Word]) OR (Verbal learning[Text Word])) OR (Spatial learning[Text Word])) OR (Learning[MeSH Terms])) OR (Verbal learning[MeSH Terms])) OR (Spatial learning[MeSH Terms])	834,623
8	(Problem solv*[Text Word]) OR (Problem solving[MeSH Terms])	43,506
9	(((Task switch*[Text Word]) OR (multitask*[Text Word])) OR (Multitasking behavior*[Text Word])) OR (Multitasking behavior[MeSH Terms])	6,307
10	((((((((((((((((((memor*[Text Word]) OR (Mental recall[Text Word])) OR (Long term memor*[Text Word])) OR (Remote memor*[Text Word])) OR (Episodic memor*[Text Word])) OR (Prospective memor*[Text Word])) OR (Short term memor*[Text Word])) OR (Spatial memor*[Text Word])) OR (Working memor*[Text Word])) OR (Immediate recall[Text Word])) OR (Psychological retention[Text Word])) OR (psychological recognition[Text Word])) OR (Memory train*[Text Word])) OR (Memory[MeSH Terms])) OR (Memory, Long-Term[MeSH Terms])) OR (Mental recall[MeSH Terms])) OR (Spatial memory[MeSH Terms])) OR (Memory, short-term[MeSH Terms])) OR (Memory, episodic[MeSH Terms])) OR (Retention, psychology[MeSH Terms])	390,339
11	1 AND 2	157
12	3 -10	2,039,819
13	(11 AND 12) Filters: English	63
14	(("Animals"[MESH] OR "Animal Experimentation"[MESH] OR "Models, Animal"[MESH] OR "Vertebrates"[MESH]) NOT ("Humans"[MESH] OR "Human experimentation"[MESH])) Filters: English	4,567,347
15	13 NOT 15	59
