# Supplementary material for: A map of evidence using transcranial direct current stimulation (tDCS) to improve cognition in adults with traumatic brain injury (TBI)
Source: Front Neuroergon. 2023 May 12;4:1170473. doi: 10.3389/fnrgo.2023.1170473 (PMC10790940; doi:10.3389/fnrgo.2023.1170473)
Supplement: Supplementary file 2 [file Data_Sheet_2.docx]

**Identification of studies via databases and registers**

**Identification of studies via other methods**

Records identified from:

Gray literature

Google Scholar

ProQuest Theses

JBI Evidence Synthesis (n=0)

JBI Evidence Implementation (n=0)

Websites (n = 5)

Neuromodec

International Modulation Society

Organisations (n = 0)

Citation searching (n = 32)

Records removed *before screening*:

Duplicate records removed (n =92)

Records marked as ineligible by automation tools (n = 0 / NA)

Records removed for other reasons (n =0)

Records identified from:

Databases (n = 572)

CINAHL (n=197)

Cochrane (81)

Embase (n=147)

Medline (n=5)

PEDro (n=5)

PsycInfo (n=43)

Web of Science (n=?)

Registers (n =20)

ClinicalTrials.gov (n=20)

**Identification**

Records excluded in Title review.

(n = 348)

Records screened.

(n = 480)

Reports not retrieved.

(n = 0)

Reports sought for retrieval.

(n = 3)

Reports sought for retrieval.

(n = 132)

Reports not retrieved.

(n = 0)

**Screening**

Reports excluded:

1. Animal Model (n = 5)
2. Clinical Trial Protocol (n = 54)
3. Conference, Poster abstract (n=9)
4. Letter to the Editor (n = 1)
5. Non-TBI or non-tDCS (n=13)
6. Review article (n=38

Reports assessed for eligibility.

(n = 132)

Reports assessed for eligibility.

(n = 3)

Reports excluded:

Not TBI 1 (n = 2)

Studies included in review.

(n = 12)

Reports of included studies.

(n = 12)

**Included**

*Adapted From:*  Page MJ, McKenzie JE, Bossuyt PM, Boutron I, Hoffmann TC, Mulrow CD, et al. The PRISMA 2020 statement: an updated guideline for reporting systematic reviews. BMJ 2021;372:n71. doi: 10.1136/bmj.n71. For more information, visit: <http://www.prisma-statement.org/>
